# Colorimetric sensing of arsenic ions in water using a mixture of *p*-quinonimine- and *p*-quinone-functionalized gold nanoparticles

**DOI:** 10.1039/d5ra08863a

**Published:** 2026-02-18

**Authors:** Sadhana Kundu, Pradip Kar

**Affiliations:** a Department of Chemistry, Birla Institute of Technology Mesra Ranchi-835215 Jharkhand India pradipkgp@gmail.com pkar@bitmesra.ac.in

## Abstract

A simple, novel, rapid, selective, and sensitive colorimetric method was demonstrated for the successful sensing of arsenic ions in water using a colloidal mixture of equal volumes (1 : 1) of separately synthesized *p*-quinonimine- and *p*-quinone-functionalized gold nanoparticles (AuNPs). The intensity of the surface plasmon resonance absorption maxima of the colloidal mixture of AuNPs was found to decrease with increasing concentrations of As(iii) ions irrespective of the pH of the solution. Using the same colloidal mixture, the sensing of As(v) was also successfully performed after chemically reducing it to As(iii) using SnCl_2_ and KI in a concentrated HCl medium. A linearity range of up to 0.035 mM was recorded for the 1 : 1 colloidal mixture of AuNPs diluted with the same volume of water at pH 7–8 towards the detection of As(iii) ions. The change in relative absorption with the change in the concentration of As(iii) ions, *i.e.*, sensitivity, was calculated to be ∼16.5 mM^−1^, and the limit of detection of ∼2.5 × 10^−3^ mM was computed from the equation 3*σ*/slope (*σ* is the standard deviation of the blank signal). The limit of detection, that is determined higher than the WHO recommended limit (0.133 × 10^−3^ mM), can be extended by pre-concentration of the water sample through simple evaporation of a larger volume of water. The response towards As(iii) ions was found to be selective over the other tested ions, such as F^−^, Cl^−^, Br^−^, I^−^, HCO_3_^−^, SO_4_^2−^, PO_4_^3−^, CO_3_^2−^, NO_3_^−^, NO_2_^−^, Ca^2+^, Mg^2+^, K^+^, Fe^2+^, Fe^3+^, Sn^2+^, Sn^4+^, Al^3+^, and Cr^3+^, and biomolecules, such as fructose, sucrose, lactose, uric acid, ascorbic acid, and dopamine. An immediate visual decolorization was also detected by naked eye for 1 mL of 10 times diluted 1 : 1 colloidal mixture of AuNPs towards 0.5 mL aqueous As(iii) solution of ∼44 × 10^−3^ mM. The sensing mechanism was proposed by considering the agglomeration of AuNPs into micro-particles due to destabilization caused by the As(iii) ions. The sensing technique was verified to have a reliable accuracy level of ∼97% ± 2% towards the detection of total As concentration in an underground water sample.

## Abstract 2

A simple, novel, rapid, selective, and sensitive colorimetric method was demonstrated for the successful sensing of arsenic ions in water using a stable aqueous colloidal mixture of *p*-quinonimine- and *p*-quinone-functionalized gold nanoparticles.

## Graphical abstract

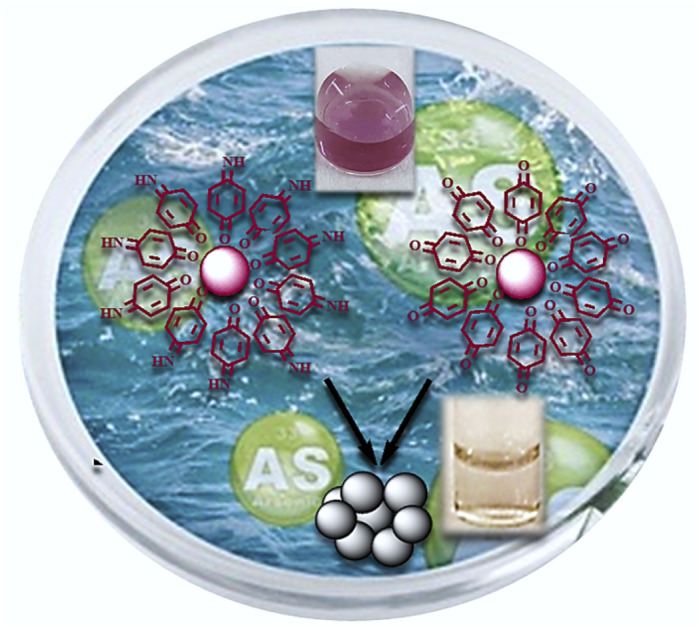

## Introduction

Over 2.5 billion individuals globally rely on groundwater as their primary source of drinking water, highlighting the critical challenge of ensuring access to clean water for human societies.^1,2^ Anthropogenic activities pose a threat to human health and environmental safety by releasing over 10 million tons of chemicals per year into water resources, which are essential to survival.^3^ There has been a notable surge in reports on arsenic contamination on a global scale, surpassing the World Health Organization's recommended limit of 10 parts per billion (ppb) or 10 µg L^−1^ or 0.133 × 10^−3^ mM in groundwater across 108 countries approximately.^3^ Prolonged exposure to arsenic-contaminated groundwater leads to severe health issues such as skin cancer, hyperkeratosis, bladder and kidney diseases, bronchiectasis, and coronary heart disease.^4,5^ In comparison to other ions, arsenic ion is unique due to its oxidation state, which governs its toxicity and solubility in aqueous media. Notably, the toxicity of inorganic arsenic varies, with As(iii) posing greater risks than As(v), and the body eliminates inorganic arsenic primarily through urine.^6^ The predominant forms of arsenic present in environmental samples are inorganic arsenite (As(iii)) and arsenate (As(v)) ions, as well as organic forms such as dithioarsenate (DTA), dimethylarsinic acid (DMA), and monomethylarsinic acid (MMA). Generally, arsenic (As) exists as both arsenite (As(iii)) and arsenate (As(v)) in natural waters or groundwater and the As(v)-to-As(iii) ratio has been found to be in the range of 10–100 depending on the pH value of water. Specifically, high levels of As(iii) (>0.3 mg L^−1^) are found in the ground water of countries like Bangladesh,^7^ India,^8^ Cambodia,^9^ and Pakistan. The standard testing methods available for those anions are expensive, laboratory-based techniques such as coupled atomic fluorescence spectroscopy (AFS), ion-selective electrodes (ISE), plasma mass spectrometry (ICP-MS), chromatography, and high-performance liquid chromatography-mass spectrometry (HPLC-MS).^10^ It is also important to develop simple procedures like colorimetric techniques for the qualitative and quantitative determination of those ions especially in aqueous media within the recommended range. The advantages of colorimetric techniques are the availability of colorimetric kits in simple forms, low cost, easy handling and using, naked eye detection, *etc.*^11,12^ Noble metal nanostructures are of great interest due to their special characteristics including major optical field enhancements that generate from high light scattering and absorption.^13^ Of these, functionalized silver and gold nanoparticles have attracted major attention from researchers due to their surface plasmon resonance (SPR) absorption maxima within the visible region. For example, cysteine- and methionine-capped silver nanoparticles have been introduced for the successful sensing of As(iii).^14^ Gold nanoparticles can be employed as very good sensing probes over silver nanoparticles due to intense visible SPR absorption maxima, sensitive SPR absorption maxima towards aggregation, more electropositive nature, easy and versatile surface functionalities, less chemical reactivity, *etc.* In order to identify arsenic in water samples, scientists have put an enormous amount of effort into creating gold nanoparticle (AuNP)-based sensors. Recently, colorimetric As(iii) sensing using localised surface plasmon resonance (LSPR) was reported for gold-modified lauryl sulphate nanoparticles with a limit of detection (LOD) of 2 ppb.^15^ The inter-particle coupling action allowed the colour change of AuNPs from pink to blue, which caused the LSPR band to shift. In another study, silk sericin-capped gold nanoparticles were used to detect As(iii) ions by UV-visible spectroscopy with an LOD of 2.94 ppb.^16^ Similarly, the colorimetric sensing performances of arsenic ions were carried out with AuNPs having several functional groups such as dithiothreitol, glutathione, citrate, cysteine, polyethylene glycol, GSH-DTT-CYs-PDCA, sucrose, and glucose.^17–24^ Most of those methods have been restricted in real applications due to the detection of As(iii) only, low sensitivity or LOD, difficult or complicated synthesis or functionalization, and the use of other modifiers or chemicals. Here, the suitable functionalization of gold nanoparticles can be carried out to make them suitable for ultrasensitive and selective colorimetric detection of both As(iii) and As(v) ions through specific interaction with the functional groups.

## Experimental

### Materials

Tetrachloroauric(iii) acid trihydrate (HAuCl_4_·3H_2_O; ≥99.0% purity) *p*-aminophenol, *p*-hydroquinone, hydrochloric acid (HCl), arsenic trioxide (As_2_O_3_), disodium hydrogen arsenate heptahydrate (Na_2_HAsO_4_·7H_2_O), stannous chloride (SnCl_2_), potassium iodide (KI) and sodium hydroxide (NaOH) pellets were acquired from Sigma-Aldrich, India. Those chemicals of synthesis grade were utilised directly without further purification. To carry out the sensing study, crystalline synthesis grade (≥99.0% purity) chemicals such as sodium salts of anions (such as fluoride, chloride, bromide, iodide, sulphate, nitrate, nitrite, bicarbonate, carbonate, and phosphate), calcium and magnesium carbonate, ferric chloride, ferrous sulphate, sucrose, fructose, lactose, ascorbic acid, uric acid, and dopamine were acquired from Sigma-Aldrich, India. Deionized water was used for the synthesis of aqueous colloids of functionalized gold nanoparticles and for all other purposes.

### Synthesis of *p*-quinonimine- and *p*-quinone-functionalized gold nanoparticles

Aqueous colloids of *p*-quinonimine- and *p*-quinone-functionalized gold nanoparticles was prepared separately following the optimized method, as reported earlier.^25,26^ First, 27 mg of tetrachloroauric(iii) acid trihydrate was dissolved in 45 ml of aqueous medium in a scratch-free two-necked round-bottomed (RB) flask with constant stirring on a magnetic stirrer. The above RB flask fitted with a water condenser on one neck was placed on a silicon oil bath to reflux the reaction mixture constantly at 80 °C throughout the duration of the reaction. Then the reduction process was conducted in two separate set-up using two reducing agents, namely, *p*-aminophenol and *p*-hydroquinone. Then, 0.05 M solution of respective reducing agent in 1 mL of 1 M aqueous sodium hydroxide diluted to 5 mL was added dropwise at a rate of 1 drop per 2 minutes from a dropping funnel attached to the other neck of the RB flask into the above reaction mixture. To keep the solution temperature constant at 80 °C throughout the reaction, continuous heat was provided to the RB. After adding the entire reagent solution, heating of the mixture at 80 °C with stirring was continued for the next 30 minutes.

### Characterizations

A Mikropack UV-vis-NIR, DH 2000 spectrophotometer was used to record the ultraviolet-visible (UV-vis) absorption spectra of diluted aqueous colloidal samples. The UV-vis spectrum was recorded for the sample in quartz cuvettes of 10 mm path length within the wavelength range from 200 to 800 nm with water as the reference. The zeta potential and size distribution histogram were recorded using a dynamic light scattering (DLS) zeta analyzer (Zetasizer Nano Series Nano-ZS, Malvern) using a very dilute aqueous colloidal sample with water acting as the reference. High-resolution transmission electron microscopic (HRTEM) images were recorded for topological characterization using a JEOL JEM 2100 PLUS instrument. To observe the images, a drop of the colloidal sample was dried on a carbon-coated copper grid (Sigma-Aldrich, India) for about 2 h at 60 °C. A Thermo Nicollet Nexus 870 instrument was used to record the Fourier transform infrared (FTIR) spectrum of a drop of colloidal sample between the wavenumber range of 4000–500 cm^−1^.

### Standard preparation

First, a 10 ppm As(iii) solution was prepared by dissolving 0.66 g of arsenic trioxide into ∼25 mL of dilute sodium hydroxide in a 500 mL volumetric flask. After making a clear solution, the volume was made up to the mark followed by the addition of 1–2 drops of dilute sodium hydroxide solution to adjust the neutral pH of the solution. Then, the solution was shaken carefully to make the solution homogeneous. Similarly, 250 mL of 10 ppm As(v) solution was prepared in a volumetric flask by dissolving 0.52 g of disodium hydrogen arsenate heptahydrate in water and making the volume up to the mark. To reduce As(v) to As(iii), 25 mL of above aqueous solution of 10 ppm As(v) was mixed with 3 mL of conc. HCl followed by the addition of 70 mg of KI and 20 mg of SnCl_2_. The reaction mixture was continuously stirred for 15 to 30 min on a magnetic stirrer at room temperature. After reduction, the concentration of As ion in the prepared solution was reduced to 5 ppm and that was confirmed by the inductively coupled plasma optical emission spectroscopy (ICP-OES) analysis with Optical 2100DV ICP-OES, Perkin Elmer. Aqueous solutions of 1000 ppm of various salts of different ions, namely, F^−^, Cl^−^, Br^−^, I^−^, HCO_3_^−^, SO_4_^2−^, PO_4_^3−^, CO_3_^2−^, NO_3_^−^, NO_2_^−^, Ca^2+^, Mg^2+^, K^+^, Fe^2+^, Fe^3+^, Sn^2+^, Sn^4+^, Al^3+^, and Cr^3+^, and biomolecules, namely, fructose, sucrose, lactose, uric acid, ascorbic acid, and dopamine were prepared by dissolving the appropriate amount in distilled water at room temperature (25 ± 2 °C).

### Sample collection and preparation

A local underground water sample was collected in a cleaned glass bottle from a deep-well hand pump in a local area with arsenic contamination, as declared by the respective governmental agency. The collected water was stored in the air-tight glass bottle at room temperature for use. In order to reduce As(v) to As(iii), first 10 mL underground water was treated with a mixture of 10 mg SnCl_2_ and 35 mg KI in 1.5 mL concentrated HCl. The experimental concentration of the As ion in the collected water sample was determined by ICP-OES analysis using a particular volume of local underground water sample.

### Colorimetric sensing study

The colorimetric sensing experiment involved recording the UV-vis spectrum of the colloidal mixture of equal volumes (1 : 1) of separately synthesized *p*-quinonimine- and *p*-quinone functionalized gold nanoparticles after the addition of a specific concentration of the analyte. This spectrum was recorded using a Mikropack UV-Vis-NIR, DH 2000 instrument at room temperature (25 ± 2 °C) across a wavelength range of 200 nm to 800 nm with water serving as the reference. As shown in Table S1, the UV-vis spectrum of 1.5 mL of 1 : 1 AuNP colloidal mixture was recorded at each step after the addition of 100 µL of 10 ppm As(iii) solution at pH 8 up to 1.9 mL. The same procedure was followed for the sensing of As(v) ions after chemically reducing it into As(iii) using the above described method. The UV-vis spectrum of 1.5 mL of 1 : 1 AuNP colloidal mixture was recorded at each step after the addition of 200 µL of above reduced solution containing 5 ppm As(iii) at pH 2 up to 1 mL (Table S1). Aqueous solutions of various interfering ionic salts or biomolecules with particular concentrations were used towards the colorimetric sensing of individual analytes or interferences for the sensing of As(iii), as shown in Table S2. The total As ion concentration in local underground arsenic-contaminated water sample was also performed for the validation of the present method, and the result was compared with the ICP-OES analysis results of the As ions.

## Results and discussion

### Formation of functionalized gold nanoparticles

**Fig. 1:**
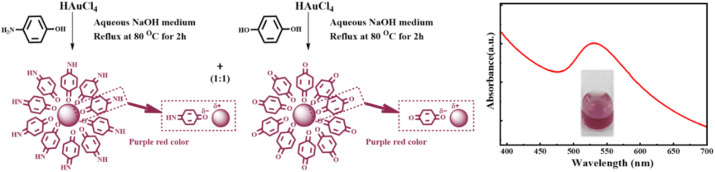
Formation mechanism and UV-vis spectrum with purple-violet visual appearance (inset) of the equal volume (1 : 1) colloidal mixture of *p*-Q- and *p*-QI-functionalized gold nanoparticles.

**Fig. 2:**
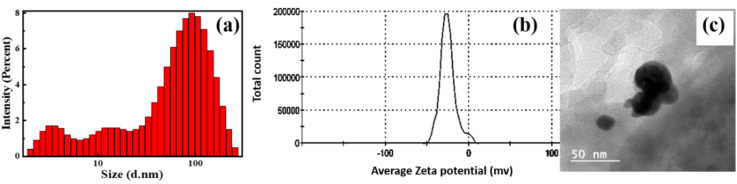
(a) Hydrodynamic size and (b) zeta potential obtained from the DLS analysis and (c) HRTEM image of the equal volume (1 : 1) aqueous colloidal mixture of *p*-Q- and *p*-QI-functionalized AuNPs.

An aqueous colloid of gold nanoparticles (AuNPs) was synthesized separately using *p*-aminophenol and *p*-hydroquinone as reducing agents and stabilizers by following the optimized method as described above. Here, *p*-aminophenol or *p*-hydroquinone in an aqueous basic medium acted as the reducing agent and stabilizer during the synthesis of AuNPs in an aqueous colloidal state similar to that of *p*-aminophenol or *o*-aminophenol.^25–27^ As established earlier,^25–27^ the Au^+3^ ion was reduced to Au(0) followed by the oxidation of *p*-aminophenol to *p*-quinonimine (*p*-QI) and *p*-hydroquinone to *p*-quinone (*p*-Q), respectively (Fig. 1). The synthesized Au(0) nanoparticles were instantly stabilized due to functionalization on the surface through electrostatic dipolar interactions with =NH functionalities of *p*-QI and =O functionalities of *p-Q*, respectively (Fig. 1) similar to that of citric acid stabilization.^28^ Both the synthesized colloids of AuNPs were appeared with a vissible color individually due to SPR absorption maxima in UV-vis spectrum at ∼540 nm (Fig. S1). In order to explain the colloidal stability of the synthesized AuNPs, the hydrodynamic size distribution and zeta potential of the synthesized AuNPs were recorded by a DLS method for the AuNPs synthesized separately (Fig. S2 and S3). The *p*-QI-functionalized AuNPs were found to have very good size distribution from 20 to 100 nm with an average diameter of 60 nm (Fig. S2). For *p*-Q-functionalized AuNPs, a wide size distribution was observed ranging from 20 to 1000 nm having an average diameter of 110 nm (Fig. S3). In general, the colloidal stability of nanoparticles is explained in terms of average zeta potential value close to ±30 to balance the attractive aggregation forces with their relative repulsive forces. The stability of both the AuNP colloids was confirmed from the average zeta potential values of −24.7 mV within the range of 0 to −50 mV for *p*-QI-functionalized AuNPs (Fig. S2) and −27.3 mV within the range of 5 to −45 mV for *p*-Q-functionalized AuNPs (Fig. S3). The size of the AuNPs was further confirmed from the TEM analysis as there are a distinct difference between the hydrodynamic size of the nanoparticle colloid and the actual size of the nanoparticles (Fig. S4). As shown in Fig. S4A, uniform and well distributed nearly perfect spherical 4-QI-functionalized AuNPs were found to have an average diameter of 10 nm. However, slightly bigger cylinder-shaped particles with an average size of 50 nm were observed in the TEM image of *p*-Q-functionalized AuNPs (Fig. S4). Very wide hydrodynamic size distribution was observed in the DLS analysis of the aqueous colloid of *p*-Q-functionalized AuNPs due to the formation of such cylinder-shaped particles. The absorption maxima in the UV-vis spectra of the mixture of aqueous colloids of *p*-QI- and *p*-Q-functionalized AuNPs appeared at 535 nm, which exhibited a vivid purple-red appearance. For evidence, Fig. 1 presents the formation mechanism and the UV-vis spectrum including visual appearance (inset) of the colloidal mixture of equal volumes (1 : 1) of *p*-Q- and *p*-QI-functionalized gold nanoparticles. The highest absorption intensity was noted for the 1 : 1 (v/v) mixture over that of the 2 : 1 or 1 : 2 colloidal mixtures of *p*-QI- and *p*-Q-functionalized AuNPs (Fig. S5). The hydrodynamic particle size distribution of the 1 : 1 colloidal mixture was obtained within the wide range from 10 to 110 nm, showing a distinct region of size distribution for the mixture (Fig. 2a). In comparison, a very poor hydrodynamic size distribution pattern was noted for either 1 : 2 or 2 : 1 (v/v) mixture of aqueous colloids of *p*-QI- and *p*-Q-functionalized AuNPs (Fig. S6). It was confirmed that the individual colloids of *p*-QI- and *p*-Q-functionalized AuNPs did not respond to the As(iii) ion. The feasibility of the colloidal mixture was tested towards the use of arsenic ion sensing application (Fig. S5). As shown in Fig. S5, a higher relative decrease in absorption intensity was noted for the 1 : 1 colloidal mixture over the 1 : 2 or 2 : 1 (v/v) mixture towards the sensing of As(iii) ions. Hence, the equal volume mixture of aqueous colloids (1 : 1) of *p*-QI- and *p*-Q-functionalized AuNPs was selected for use in the study of colorimetric sensing performances towards As ions. The stability and morphology of the 1 : 1 colloidal mixture of *p*-QI- and *p*-Q-functionalized AuNPs were further characterized from DLS zeta potential and TEM analysis. A zeta potential value was obtained as −25.3 mV (Fig. 2b) to explain the sufficient stability of this 1 : 1 colloidal mixture for further applications.^29^ The dark-field TEM images revealed the existence of two distinct mixtures of nanoparticles with average diameters of ∼14 nm for *p*-QI-functionalized AuNPs and ∼46 nm for *p*-Q-functionalized AuNPs (Fig. 2c).

### UV-vis spectroscopic sensing performances towards arsenic ions

**Fig. 3:**
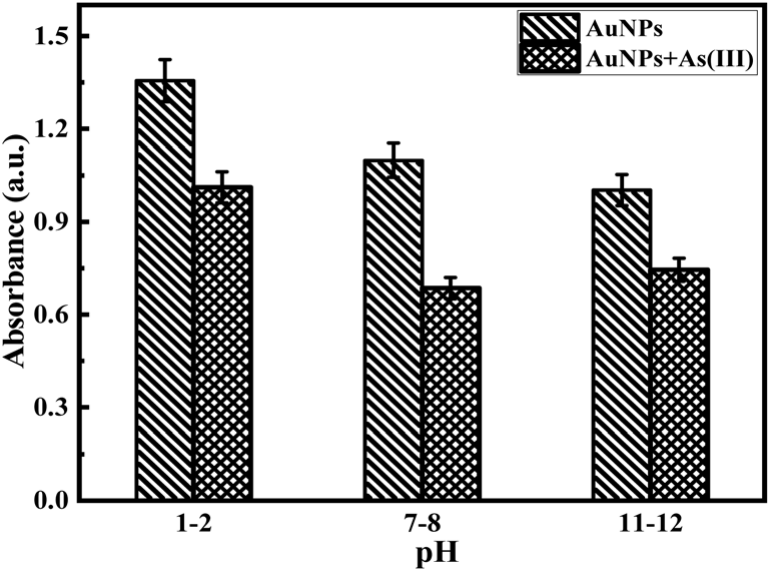
Comparison of the absorption intensity at ∼530 nm in the UV-vis spectra of the equal volume (1 : 1) AuNP colloidal mixture at pH 1–2, 7–8, and 11–12 before and after the addition of aqueous As(iii) ion solutions.

**Fig. 4:**
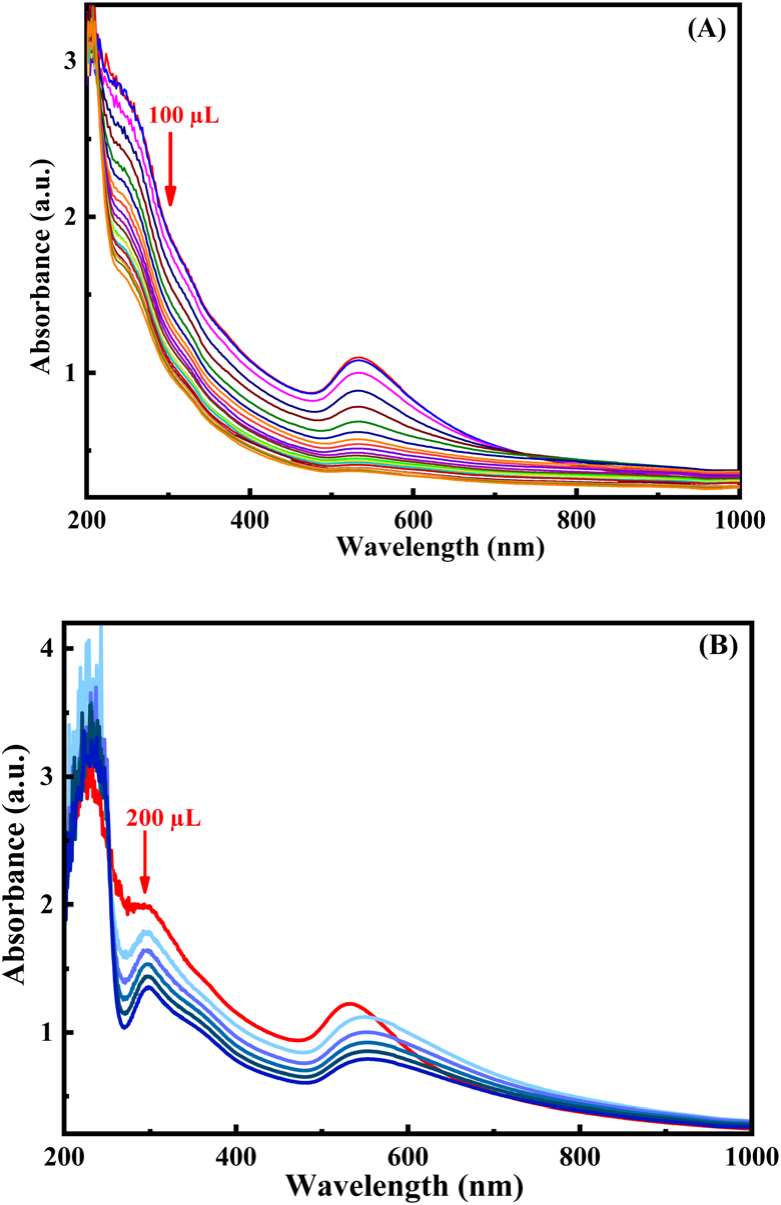
UV-vis spectra of 1.5 mL equal volume (1 : 1) AuNP colloidal mixture at pH 7–8 diluted to 3 mL with the stepwise addition of a (A) 100 µL aqueous solution of 10 ppm As(iii) ions and (B) 200 µL aqueous solution of 10 ppm As(v) ions chemically reduced to As(iii).

The relative decrease in the absorption intensity of the equal volume or 1 : 1 (v/v) colloidal mixture of *p*-QI- and *p*-Q-functionalized AuNPs towards different concentrations of aqueous solutions of arsenic ions was determined from the UV-vis absorption spectrum at room temperature (25 ± 2 °C). It is well known that the decrease in absorption intensity is directly proportional with the decrease in the concentration of the respective solution as per Beer–Lambert's law. For the verification of the dilution effect on the absorption intensity of AuNP colloidal mixtures, the UV-vis spectra (Fig. S7) of particular volumes of the colloidal mixture were recorded with step-wise addition of 1 mL water up to 5 mL dilution. The intensity of the characteristic absorption intensity at ∼530 nm of the 1 : 1 (v/v) colloidal mixture of *p*-QI- and *p*-Q-functionalized AuNPs was gradually decreased with water dilution (Fig. S7). As shown in Fig. S7, the decreasing trend of absorption intensity was found to be significantly low towards the addition of 10 ppm As(iii) ions in comparison to the water dilution. In order to know the influence of pH on the sensing response towards As(iii) ions, the change in the absorption intensity of the colloidal mixture was recorded by UV-vis characterization towards the addition of 500 µL of 10 ppm As(iii) ions (Fig. 3). The change in the SPR absorption maxima in the UV-vis spectra of the colloidal mixture of AuNPs at different pH values of the medium is shown in Fig. S8. A slight decreasing trend was observed in the intensity of absorption maxima of the 1 : 1 (v/v) colloidal mixture with the increase in pH from 2 to 12 (Fig. S8). This is due to the lack of interactions, especially hydrogen bonding interactions between the *p*-QI and *p*-Q functionalities at the acidic pH of 2 due to protonation on the =NH or =O functional groups.^25^ The above-mentioned interactions are considered to be strong at the neutral to basic pH for weak negative polarity on the =NH or =O functional groups of *p*-QI and *p*-Q, respectively.^25^ However, the intensity of absorption maxima of the colloidal mixture was altered, nearly identical to the addition of 500 µL aqueous solution of 10 ppm As(iii) ions at pH 2, 8 or 12 (Fig. 3). Hence, it can be concluded that the medium pH was found to have a little influence on the stability of the 1 : 1 (v/v) colloidal mixture of *p*-QI- and *p*-Q-functionalized AuNPs, whereas the sensing performances of the mixture towards As(iii) ions was not significantly influenced by the pH of the medium. The variation in intensity for the characteristic SPR absorption maxima of the AuNP mixture at pH 7–8 in the UV-vis spectra upon the step-wise addition of 10 ppm As(iii) ions is shown in Fig. 4A. A significant and gradual decrease in the intensity of the absorption maxima at ∼530 nm was observed in the UV-vis spectra of the AuNP mixture at pH 8 towards the increasing concentration of As(iii) ions. It was noted that the aqueous solution of As(v) ions did not respond by decreasing the absorption intensity in the UV-vis spectra of the AuNP mixture.^30^ Hence, the As(v) ions were chemically reduced to As(iii) ions by using SnCl_2_ and KI mixture in a HCl medium, and then the sensing response was studied following the same procedure used for As(iii) ions. A significant decrease in absorption intensity was also observed in the UV-vis spectra of the AuNP mixture with the increase in the concentration of As(v) ions chemically reduced to As(iii) ions (Fig. 4B).

### Visual sensing performances towards arsenic ions

**Fig. 5:**
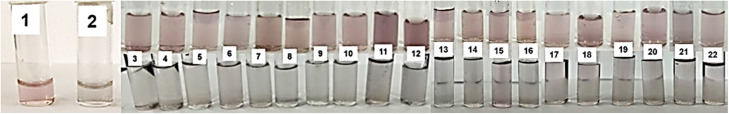
Representation of visual color (purple-red) variation upon the addition of (1) 0.5 mL water, (2) 0.5 mL 10 ppm As(iii), 0.5 mL 1000 ppm aqueous solution of anions [(3) F^−^, (4) Cl^−^, (5) Br^−^, (6) I^−^, (7) HCO_3_^−^, (8) SO_4_^2−^, (9) PO_4_^3−^, (10) CO_3_^2−^, (11) NO_3_^−^, and (12) NO_2_^−^,], cations [(13) Ca^2+^, & Mg^2+^, (14) K^+^, (15) Fe^2+^, and (16) Fe^3+^], and biomolecules [(17) fructose, (18) sucrose, (19) lactose, (20) uric acid, (21) ascorbic acid, and (22) dopamine] in equal volume (1 : 1) AuNP colloidal mixture. The images at the bottom show the decolorization of each of the above mixture upon the addition of 0.5 mL of 10 ppm As(iii) solution.

The visual sensing performances towards the arsenic ions and the interference effect of other common anions, cations, and biomolecules are shown in Fig. 5. As shown in Fig. 5, the color change of the 0.5 mL 1 : 10 (v/v) diluted AuNP colloid is recorded visually at RT after the addition of 0.5 mL of 10 ppm As(iii) solution. It should be noted that with dilution, the detection of visual color was found to become difficult, and hence, the dilution of the AuNP colloidal mixture must be kept at 1 : 10 (v/v) for the sensing study. To check the effect of dilution on the visual color change of the AuNP mixture, the blank test was also performed by adding 0.5–1.0 mL distilled water in 0.5 mL AuNP colloid. A very good purple-red color was visualized for the blank test due to the absorption maxima of the mixture of AuNP colloids at 535 nm. Upon adding a certain concentration of As(iii) ions, the mixture of functionalized AuNP colloids showed a rapid selective decolourization from purple-red (Fig. 5). This type of decolorization was not observed for the addition of same concentrations of ions, namely, F^−^, Cl^−^, Br^−^, I^−^, HCO_3_^−^, SO_4_^2−^, PO_4_^3−^, CO_3_^2−^, NO_3_^−^, NO_2_^−^, Ca^2+^, Mg^2+^, K^+^, Fe^2+^, and Fe^3+^ and biomolecules, namely, fructose, sucrose, lactose, uric acid, ascorbic acid, and dopamine with the particular volume of a mixture of functionalized AuNP colloids (Fig. 5), whereas an immediate decolorization of the mixture of functionalized AuNP colloids was observed from purple-red by the simple addition of As(iii) ions into the colloidal mixture in the presence of the other ions. Hence, it can be said that the above-tested ions/biomolecules were not found to have any effect on the visual color change of the mixture of functionalized AuNP colloids. Those ions/biomolecules did not have any interference in the visual detection of the As(iii) ions by the mixture of functionalized AuNP colloids.

### Analytical figure of merits

**Fig. 6:**
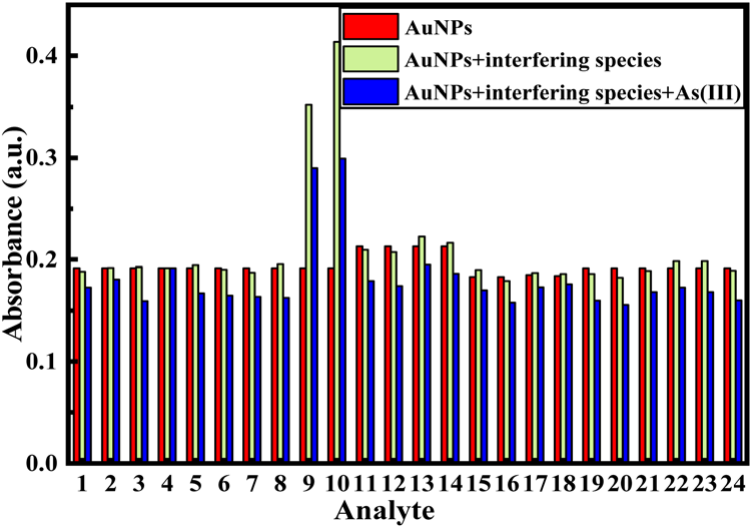
Graphical representation of the change in the absorbance value of equal volume (1 : 1) AuNP colloidal mixtures upon the addition of aqueous solutions of anions [(1) F^−^, (2) Cl^−^, (3) Br^−^, (4) I^−^, (5) HCO_3_^−^, (6) SO_4_^2−^, (7) PO_4_^3−^, (8) CO_3_^2−^, (9) NO_3_^−^, and (10) NO_2_^−^,], 0.5 mL of cations [(11) Ca^2+^, Mg^2+^, (12) K^+^, (13) Fe^2+^, (14) Fe^3+^, (15) Sn^2+^, (16) Sn^4+^, (17) Al^3+^ and (18) Cr^3+^] and 0.5 mL of biomolecules [(19) fructose, (20) sucrose, (21) lactose, (22) uric acid, (23) ascorbic acid, and (24) dopamine], followed by the addition of an aqueous As(iii) solution.

In order to observe the selective responses towards the As(iii) ions over the ions, namely, F^−^, Cl^−^, Br^−^, I^−^, HCO_3_^−^, SO_4_^2−^, PO_4_^3−^, CO_3_^2−^, NO_3_^−^, NO_2_^−^, Ca^2+^, Mg^2+^, K^+^, Fe^2+^, Fe^3+^, Sn^2+^, Sn^4+^, Al^3+^, and Cr^3+^, and biomolecules, namely, fructose, sucrose, lactose, uric acid, ascorbic acid, and dopamine, including their interference effect, the UV-vis spectrum of the colloidal mixture was recorded after the addition of particular concentrations of ions or molecules with/without As(iii) ions (Table S1). The graphical representation of the change in the maximum absorbance value of a mixture of AuNPs upon addition of aqueous solutions of various ions or biomolecules and then addition of an As(iii) solution is depicted in Fig. 6. As shown in Fig. 6, the absorption intensity of the colloidal mixture remained unchanged upon the addition of all the tested anions, cations and biomolecules except nitrate/nitrite and iodide ions. The absorption intensity of a colloidal mixture in the presence of nitrate/nitrite ions was found to increase probably due to the inherent colour of these two ions in their aqueous solution (Fig. 6). The absorption intensity of the colloidal mixture decreased as usual and almost identically within the range between 15 and 18% towards the addition of As(iii) ions of the same concentration in the presence of all the ions or biomolecules except iodide. The absorption intensity was noted to be unaffected in the presence of iodide ions probably due to the violet colour of iodine itself. Therefore, it can be concluded that iodide with a sufficient high concentration is supposed to show interference in the sensing of As(iii) ions, though other tested ions/biomolecules are not found to have any interference effect at least at very low concentrations.

**Fig. 7:**
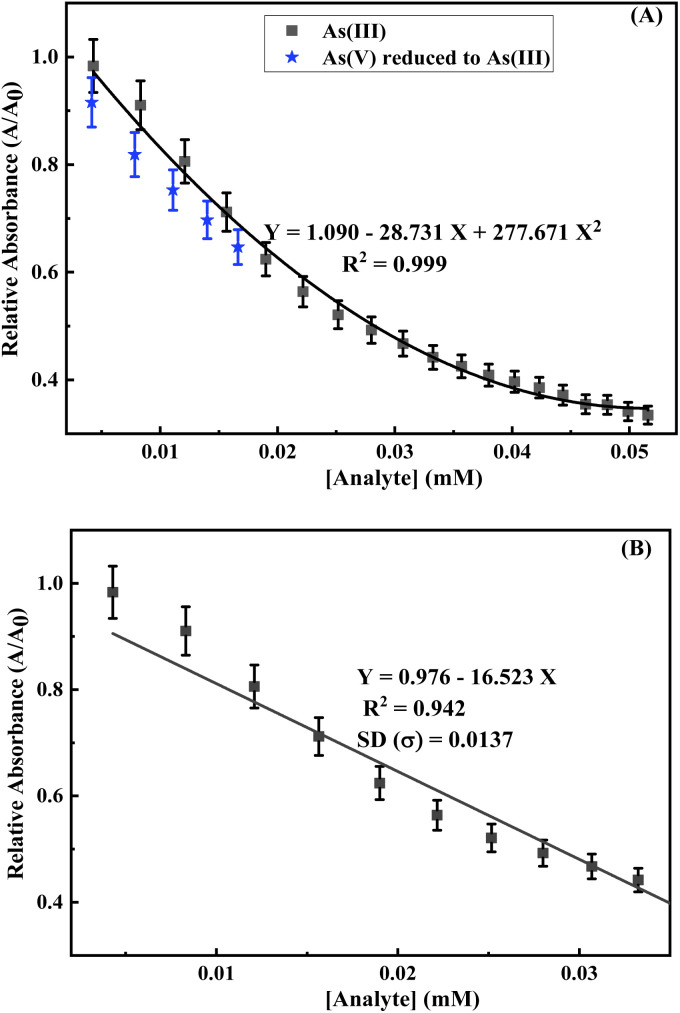
(A) Change in the relative absorption (*A*/*A*_0_) at 530 nm for different concentrations of As(iii) or As(v) reduced to As(iii). (B) Linear fitting calibration plot within the linear range for the change in relative absorption (*A*/*A*_0_) at 530 nm for different concentrations (in mM) of As(iii) ions.

The calibration curve was plotted to show the change in the relative absorbance of the mixture of AuNPs with the change in analyte concentration (Fig. 7A). A very good second-degree polynomial curve of the change in the relative absorbance of the equal volume colloidal mixture of AuNPs diluted with same volume of water at pH 7–8 with the change in the concentration of the aqueous solution of As(iii) ions was fitted to have a correlation coefficient (*R*^2^) value of 0.99. The change in the intensity of absorption maxima for the same concentrations of AuNP mixtures was observed as little lower for the relative absorbance of the mixture of AuNPs with different concentrations of the aqueous solution of As(v) ions chemically reduced to As(iii) ions (Fig. 7A). This could be due to the little dilution effect towards the addition of a higher volume of chemically reduced As(v) solution than that of the As(iii) solution. A linearity range was noted from 0.0042 mM to 0.035 mM concentration of As(iii) ion for sensing by the equal volume colloidal mixture of AuNPs diluted with the same volume of water at pH 7–8, and the corresponding linear fitting equations (*R*^2^ value of 0.94) are also included in Fig. 7B. A sensitivity in terms of the change in relative absorption with the change in the concentration of analytes was calculated in terms of the slope of the linear fitting equation as ∼16.5 mM^−1^. The limit of detection (LOD) defined as 3*σ*/slope (*σ* is the standard deviation of the blank signal) was also calculated from the calibration plot as ∼2.5 µM for As(iii) ions. It should be noted that the limit of detection was determined to be about 20 times higher than that of the WHO recommended limit (0.133 × 10^−3^ mM). However, a high volume of water sample after concentrating by evaporation should be used to extend the limit of detection below the WHO recommended limit. An immediate visual color change from purple-red to colorless was noted upon the addition of 0.5 mL of ∼44 µM aqueous As(iii) solution in 0.5 mL of 10 times diluted equal volume mixture of AuNP colloids at pH 7–8. Therefore, the visual limit of detection for the arsenic ion is considered as ∼44 µM. The limit of quantification (LOQ) is defined as the lowest measurable concentration of the analyte at 10 × *σ* with precision, *i.e.,* LOQ = 10 × *σ*/slope. Using the equation, an LOQ of 8.29 µM was calculated for the sensing of As(iii) ions by the AuNP colloidal mixture.

**Fig. 8:**
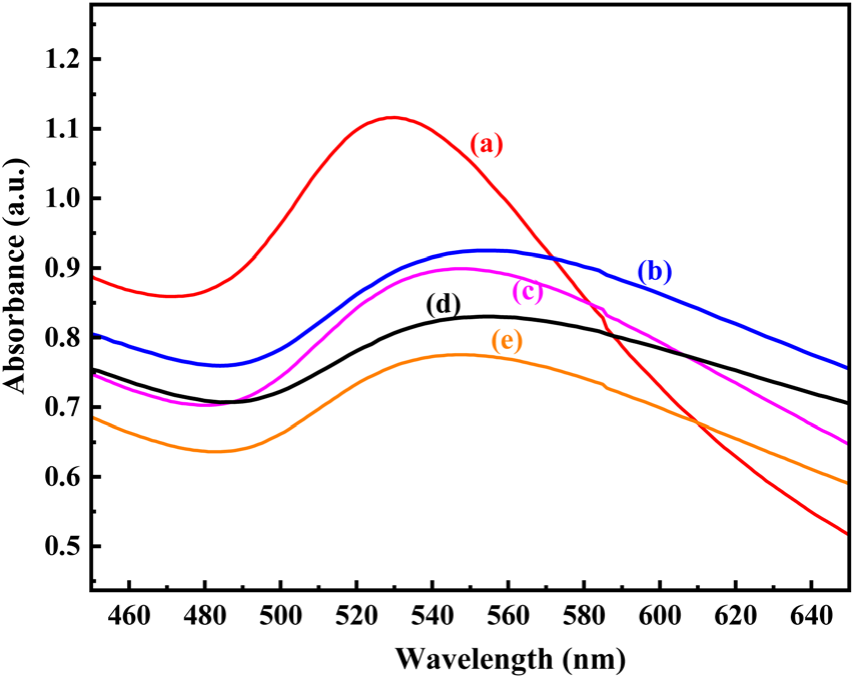
UV-vis spectrum of (a) 1.5 mL equal volume AuNP colloidal mixture diluted to 3 mL at pH 8 and after the addition of (b) 0.5 mL blank solution, (c) 0.5 mL groundwater sample, (d) 1 mL blank solution, and (e) 1 mL groundwater sample.

In order to verify the efficiency of the equal volume mixture of AuNPs towards the sensing of As ions in real applications, the study was performed using a raw local underground water sample contaminated with As ions. The blank study was performed with uncontaminated tap water keeping in mind that the dilution might have some influence on the absorption intensity of the AuNP mixture (Fig. S9). The concentration of As ion in local underground water sample was calculated by comparing the absorption intensity at ∼530 nm with that of blank test. The comparison of the experimental As ion concentration from the ICP-OES analysis with the calculated value using the calibration formula, as shown in Fig. 7B, is shown in Table S2. As per the ICP-OES analysis, the real water sample was contaminated with 0.35 ± 0.1 mg L^−1^ As ions. From Fig. S9 and the calculated concentration of As shown in Table S2, it was clear that 0.5 to 1.2 mL of real water sample should be used to obtain the concentration with less than 10% error. The use of even a smaller volume of water sample was found to produce results with an error of 46–55% due to the difficulty in measuring minute concentrations, while the use of a larger volume of water was restricted by very weak absorption intensity. The best result using 0.5 and 1 mL of real water sample is shown in Fig. 8, including the calculated value shown in Table 1. A very high percentage of accuracy within the range of 97% ± 2% was obtained for the sensing of As ions in the underground water sample, considering the upper value as standard (Table 1). This indicated the usability, reliability, effectiveness, and efficiency of the colorimetric sensing of arsenic ions in water using a mixture of *p*-Q- and *p*-QI-functionalized gold nanoparticles.

**Table 1:** Comparison of the experimental As ion concentrations obtained *via* ICP-OES with the calculated value obtained *via* the calibration formula

Sample	ICP-OES experimental amount of As (mg L^−1^)	Vol (mL)	Calculated amount of As (mg L^−1^)	% accuracy or recovery
As ion-contaminated underground water	0.35 ± 0.1 (considering upper value of 0.45)	0.5	0.442	98
		1	0.430	95

### Mechanism of arsenic sensing: the chemical interpretation

**Scheme 1:**
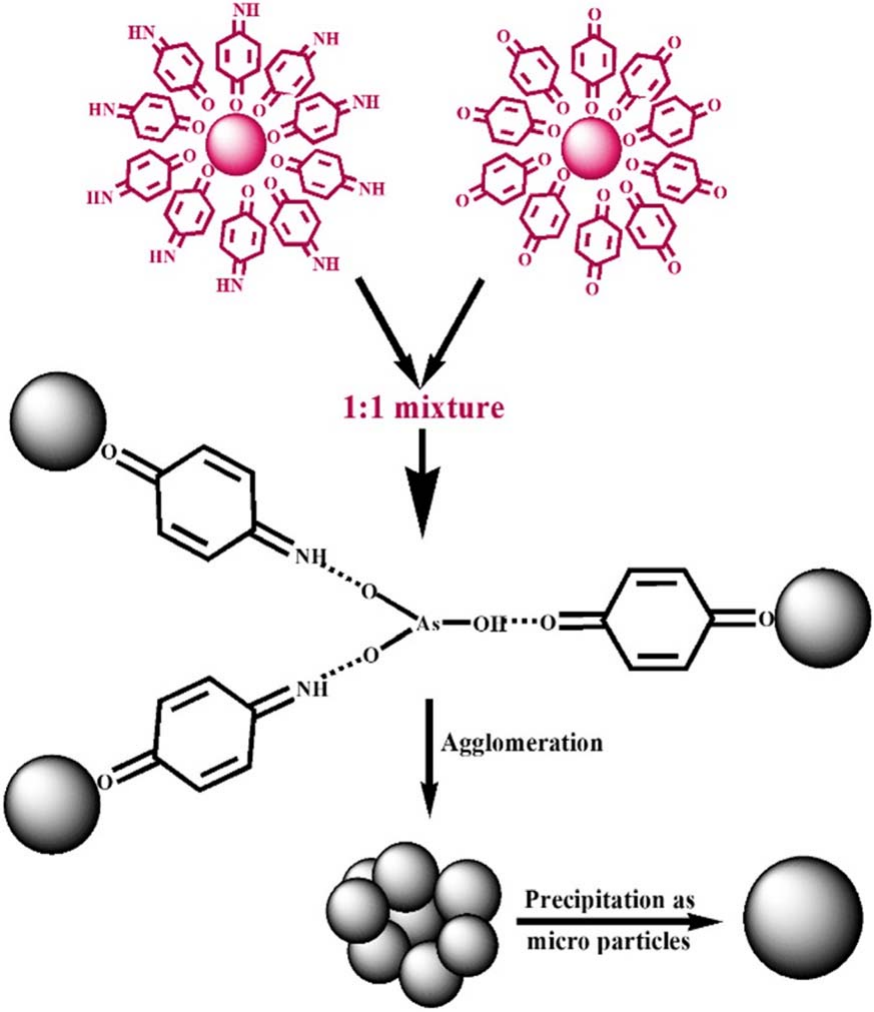
Proposed sensing mechanism of As(iii) ions by the AuNP colloidal mixture.

**Fig. 9:**
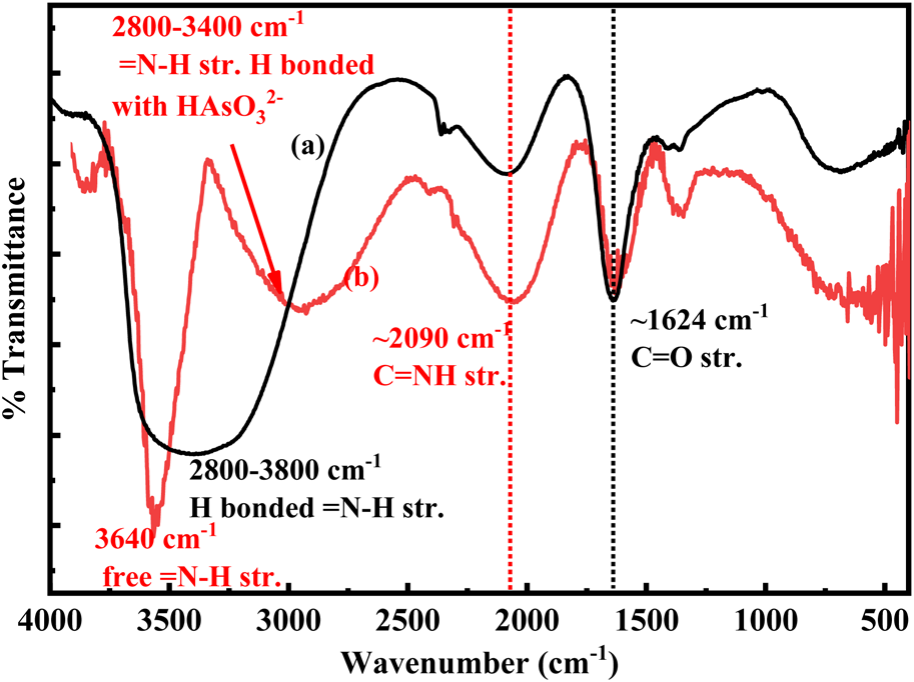
FTIR spectra of the colloidal mixture of AuNPs (a) before and (b) after the addition of As(iii) ions.

The proposed mechanism for the sensing of arsenite ions using 1 : 1 (v/v) colloidal mixture of *p*-QI- and *p*-Q-functionalized AuNPs is shown in Scheme 1. The functionalization of *p*-QI and p-quinone by dipolar interactions of their electronegative =O groups with the electropositive surface of AuNPs stabilised the AuNPs in the aqueous colloidal form, as shown in Fig. 9 and Scheme 1. The dipolar interaction between the *p*-QI and *p*-Q functional groups and the AuNPs was supposed to break up upon the addition of As(iii) ions, which triggered the AuNPs to aggregate and precipitate out as microparticles. This is because the highly electronegative oxyanion of arsenite formed strong hydrogen bonding with the =N–H functional groups of the *p*-QI stabilizer from one side and the other hydrogen bonding formed between the –OH group of arsenite and the =O group of the quinone stabilizer from the other side. Hence, the intensity of the absorption maxima decreased with increasing As(iii) ion concentration, and then disappeared above a particular concentration . This type of simultaneous interaction with both =O and =N–H groups should not be possible for the individual colloids of *p*-QI- and *p*-Q-functionalized AuNPs towards the As(iii) ion. Therefore, the individual colloids of *p*-QI- and *p*-Q-functionalized AuNPs were found to have no response towards the As(iii) ion. As per Scheme 1, the *p*-QI-functionalized AuNPs and *p*-Q-functionalized AuNPs in a stoichiometric ratio of 2 : 1 were interacting with HAsO_3_^2−^ to initiate the agglomeration. Still, the 1 : 1 colloidal mixture was found to have a better sensing response to As(iii) ions over the 1 : 2 or 2 : 1 (v/v) mixture, as evident earlier (Fig. S5). The fact might be correlated by considering the higher agglomeration ability of bigger *p*-Q-functionalized AuNPs than that of the smaller *p*-QI-functionalized AuNPs. The mechanism was clearly defined by the UV-vis and DLS (Table 2) analyses. Apart from the SPR absorption maxima appearing at 535 nm in the UV-vis spectra of AuNP colloids, two other weak bands were observed at 218 nm and 302 nm. These bands corresponded to the π → π* transition of the functional group and the –O- group of *p*-QI and *p*-Q assembled on AuNPs.^31^ The *p*-QI and *p*-Q functionalities were detached from the Au surface due to the strong hydrogen bonding between the electronegative oxyanion of As(iii) and the =N–H functional groups of the *p*-QI stabilizer from one side and the other hydrogen bonding formed between the –OH group of arsenite and the =O group of the quinone stabilizer from the other side. The shifting of vibrational modes associated with these groups could also be detected from the FTIR spectrum analysis (Fig. 9).^32^ As shown in Fig. 9, the stretching bands of C=NH appearing at ∼2090 cm^−1^ and C=O at ∼1624 cm^−1^ in the FTIR spectrum of the AuNP colloidal mixture were further broadened due to hydrogen bonding with the As(iii) ions.^33,34^ In addition, the broad band in the region from 2800 to 3800 cm^−1^ for the stretching of hydrogen bonded =N–H was separated into two bands; one in the region from 2800 to 3400 cm^−1^ and other at 3640 cm^−1^ (Fig. 9). The first one is assigned to the stretching vibration of =N–H groups strongly hydrogen bonded with oxyanion of As(iii) and the other one is assigned to the stretching vibration of free =N–H groups (Fig. 9). These inferences could be well explained from the DLS analysis data, as shown in Table 2. The average zeta potential value of −25.3 mV at a boundary value of ±30 indicated the stability of the aqueous colloid of *p*-QI-functionalized AuNPs at pH 7, and the corresponding average hydrodynamic size of the AuNPs was measured as 60.86 nm (Table 2). With the increase in As(iii) ion concentration, the aqueous 1 : 1 colloidal mixture of gold nanoparticles functionalized with *p*-quinone and *p*-quinonimine eventually became unstable. As a result, when the volume of 10 ppm As(iii) at pH 8 was increased from 250 µL to 500 µL, the average hydrodynamic size increased from 175 nm to 402.91 nm (Table 2). In addition, the hydrodynamic size distribution of the AuNP mixture was obtained within region from 30 nm to 900 nm upon the addition of 250 µL of 10 ppm As(iii) solution (Fig. S10). This size distribution was agglomerated into a bigger size ranging from 200 to 600 nm due to the addition of 500 µL of 10 ppm As(iii) solution (Fig. S10). Similarly, the negative zeta potential value of the AuNP mixture was altered from 40–50 mV to 50–60 mV with the increase in the volume of 10 ppm As(iii) solution from 250 µL to 500 µL (Fig. S11).

**Table 2:** DLS analysis of 4-QI-functionalized AuNP colloids with the addition of analytes

Analyte in colloid of 4-QI-functionalized AuNPs (mL)	Water	10 ppm As(iii) pH 7–8	
	1 mL	250 µL	500 µL
Zeta potential (mV)	−25.3	−0.348	0.0702
Average hydrodynamic size (nm)	60.86	175.70	402.91

## Conclusions

In conclusion, a colorimetric sensor for the selective detection of arsenite ions in an aqueous medium using an aqueous colloidal mixture of *p*-QI- and *p*-Q-functionalized AuNPs was reported here. Because of its SPR absorption maxima at 535 nm, both the stable aqueous colloids of *p*-QI- and *p*-Q-functionalized AuNPs appeared intense purple-red in colour, detectable with the naked eye. The best UV-vis sensing response towards As(iii) ions was obtained for the equal volume mixture of the two aqueous colloids at pH 7–8. A very similar decreasing pattern of the intensity of absorption maxima was observed at pH 7–8 with respect to As(v) chemically reduced to As(iii) using SnCl_2_ and KI mixture in a HCl medium. A linearity range up to 0.035 mM with a sensitivity of ∼16.5 mM^−1^ and a limit of detection of ∼2.5 µM was observed for the detection of As(iii) ions. Here, the limit of detection was found to be 20 times higher than that of the WHO recommended limit (0.133 × 10^−3^ mM). However, it can be extended by concentrating the As ions in a large volume of water sample simply by evaporation. The response was found to be selective as well as non-interfering with respect to the ions, namely, F^−^, Cl^−^, Br^−^, I^−^, HCO_3_^−^, SO_4_^2−^, PO_4_^3−^, CO_3_^2-^, NO_3_^−^, NO_2_^−^, Ca^2+^, Mg^2+^, K^+^, Fe^2+^, Fe^3+^, Sn^2+^, Sn^4+^, Al^3+^, and Cr^3+^, and biomolecules, namely, fructose, sucrose, lactose, uric acid, ascorbic acid, and dopamine. A selective naked-eye detection of As(iii) ions was also possible above an LOD of ∼44 µM. The efficiency of the AuNP mixture was verified with a high accuracy of 97% ± 2% for the sensing of As ions in an underground water sample. Thus, the method might be explored for the on-time monitoring of As contamination in various areas of applications including domestic, municipal, agricultural, medicinal, industrial, and biological fields.

## Author contributions

Sadhana Kundu: formal analysis, data curation, methodology, investigation, writing – original draft. Pradip Kar: conceptualization, methodology, supervision, validation, writing– review & editing.

## Conflicts of interest

The authors declare that they have no known competing financial interests or personal relationships that could have appeared to influence the work reported in this paper.

## Supplementary Material

RA-016-D5RA08863A-s001

## Data Availability

The authors confirm that all the data supporting the findings of this study are available within the article and its supplementary information (SI). Supplementary information: tables with experimental conditions, characterization data and sensing plots. See DOI: https://doi.org/10.1039/d5ra08863a.
