# Supplementary material for: Colorimetric sensing of arsenic ions in water using a mixture of p-quinonimine- and p-quinone-functionalized gold nanoparticles
Source: RSC Adv. 2026 Feb 18;16(11):9733–42. doi: 10.1039/d5ra08863a (PMC12914506; doi:10.1039/d5ra08863a)
Supplement: RA-016-D5RA08863A-s001 [file RA-016-D5RA08863A-s001.pdf]

Supplementary Information (SI) for RSC Advances.
This journal is © The Royal Society of Chemistry 2026

Colorimetric sensing of arsenic ion in water using a mixture of p-quinone and p-quinonimine functionalized gold nanoparticles

Sadhana Kundu and Pradip Kar*

Department of Chemistry, Birla Institute of Technology Mesra, Ranchi-835215, Jharkhand, India

Supporting Information

Table S1. Experimental condition used to record the absorption intensity of AuNPs mixture at wavelength 530 nm towards the sensing study of As ions

Vol. of Au mixture (mL)	Water added (mL)	Vol. of analyte added (mL)
1.5 (1:1)	1.5	Stepwise 100 μL of aqueous arsenic trioxide solution in sodium hydroxide with 10 ppm As(III) solution
1.5 (1:1)	1.5	Stepwise 200 μL of aqueous disodium hydrogen arsenate solution with As(V) solution reduced to 5 ppm As(III)
0.25	2.25	50 μL of 5000 ppm aqueous solution of various ions or biomolecules
0.25	2.25	Addition of 0.5 mL aqueous arsenic trioxide solution in sodium hydroxide with 10 ppm As(III) solution in presence of 50 μL of 5000 ppm aqueous solution of various ions or biomolecules

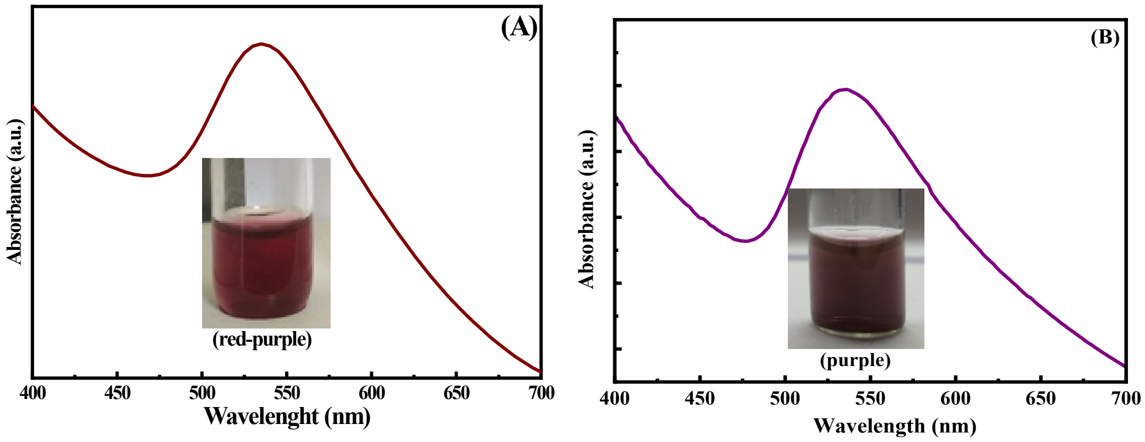

Fig. S1. UV-VIS spectrum with visual appearance (inset) of aqueous colloid of (A) p-quinonimine functionalized gold nanoparticles and (B) p-quionone functionalized gold nanoparticles

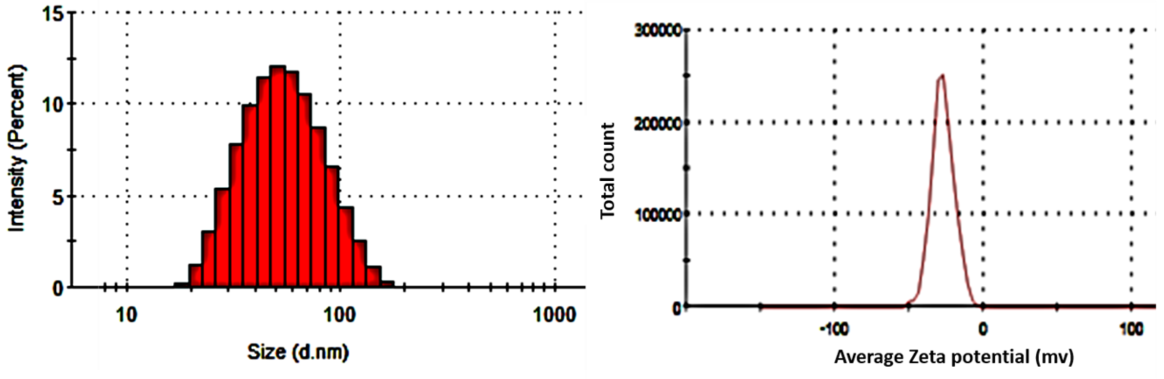

Fig. S2. Hydrodynamic size distribution (right) and zeta potential (left) obtained from DLS analysis for aqueous colloid of p-quinonimine functionalized gold nanoparticles

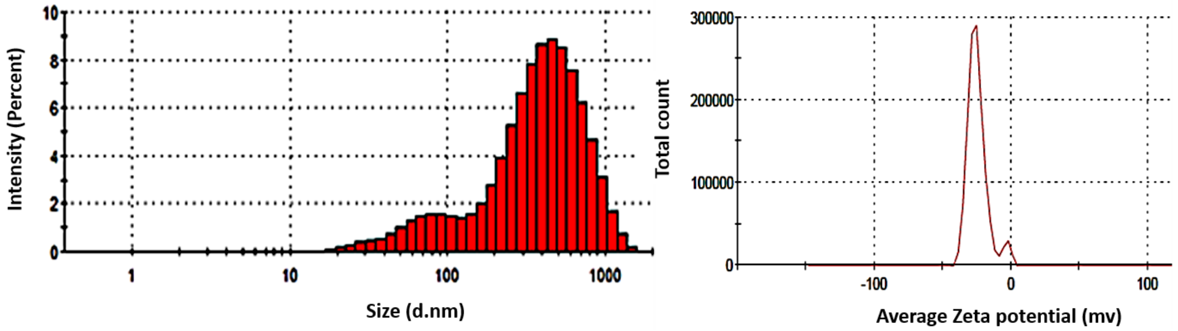

Fig. S3. Hydrodynamic size distribution (right) and zeta potential (left) obtained from DLS analysis for aqueous colloid of p-quinone functionalized gold nanoparticles

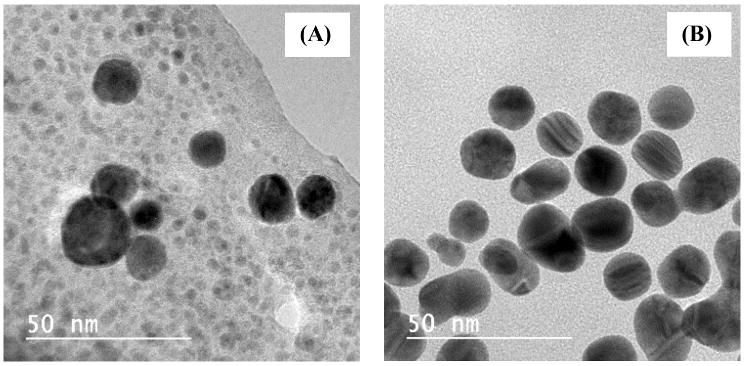

Fig. S4. HRTEM images of (A) p-quinonimine functionalized gold nanoparticles and (B) p-quionone functionalized gold nanoparticles

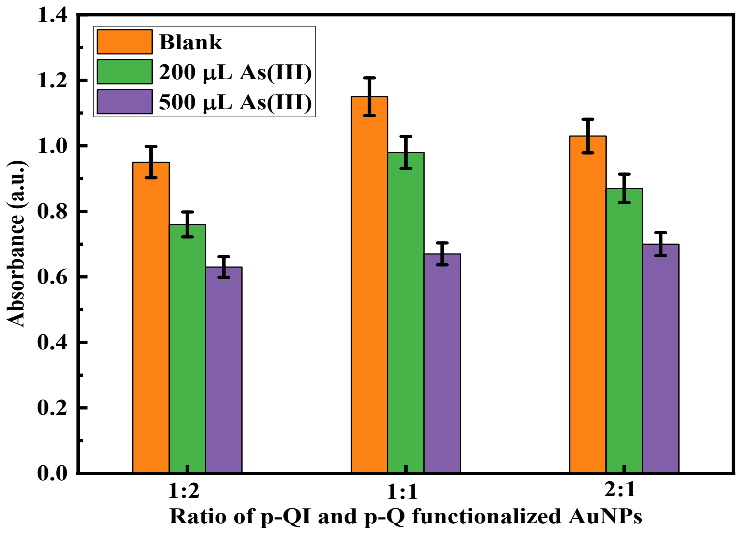

Fig. S5. The absorption intensity of aqueous colloidal mixture of p-quinonimine (p-QI) and p-quinone (p-Q) functionalized AuNP and relative decrease in absorption intensity towards 10 ppm As(III) ions

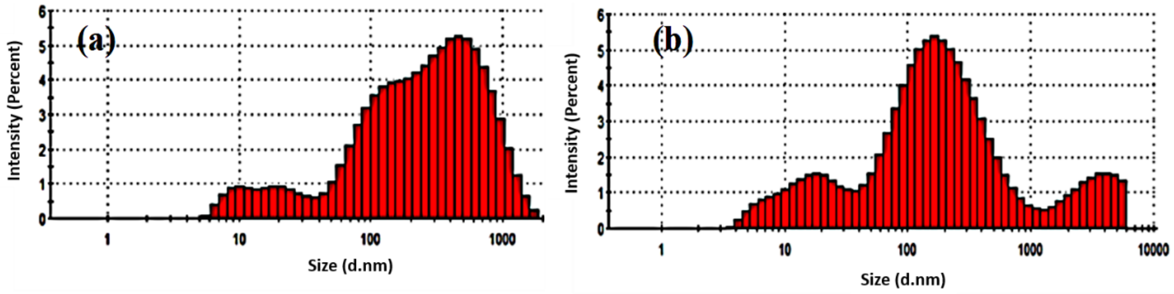

Fig. S6. Hydrodynamic size obtained from DLS analysis for (a) 1:2 and (b) 2:1 aqueous colloidal mixture of p-quinonimine and p-quinone functionalized AuNP

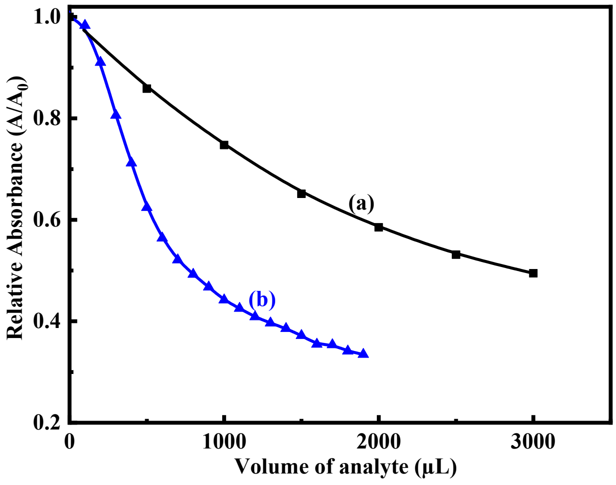

Fig. S7. UV–VIS spectra of 2.5 mL equal volume aqueous colloidal mixture of AuNPs diluted to 5 mL towards stepwise addition of (a) water only and (b) water with 10 ppm As(III) ion

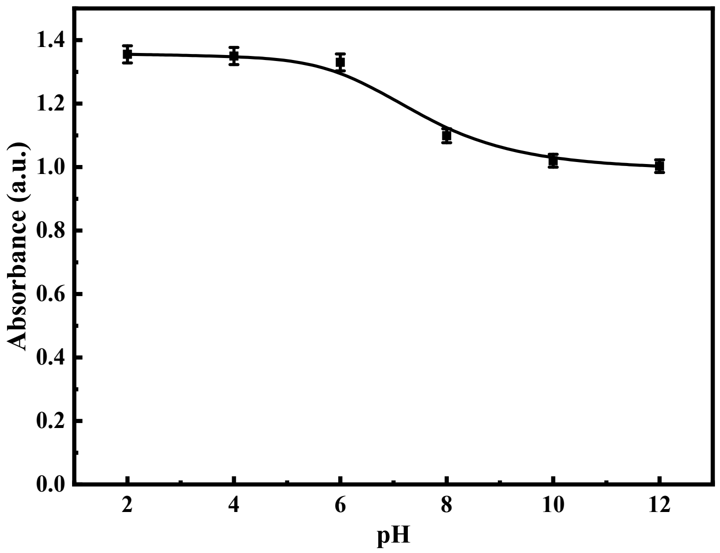

Fig. S8. Change of SPR absorption maxima in UV–VIS spectra of colloidal mixture of AuNPs with varying pH of the medium.

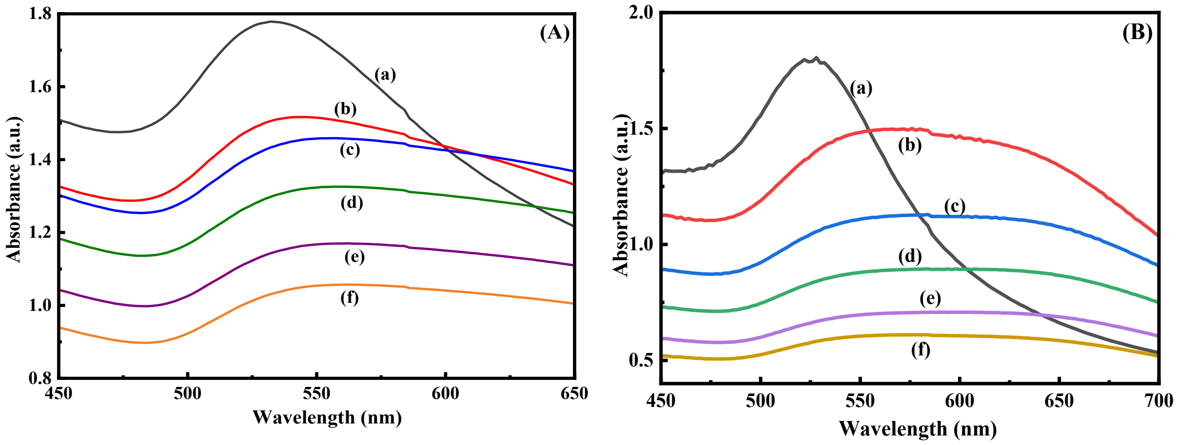

Fig. S9. UV-VIS response of colloidal mixture of AuNPs towards (A) blank sample and (B) arsenic contaminated real water sample of (a) 0, (b) 0.2, (c) 0.3, (d) 0.5, (e) 1, (f) 1.2 mL

Table S2. Experimental condition to study the sensing response of colloidal mixture of AuNPs towards blank sample and arsenic contaminated real water sample

Vol. of 1:1 AuNPs mixture used diluted up to 3 mL (mL)	Vol. of real water sample used (mL)	Absorption intensity @ 530 nm (a.u.) (Fig. S10)		[As] of repeating for 3 times (mg L^{-1})	% Error of repeating for 3 times	% Accuracy with respect to upper value of 0.45 mg L^{-1}
		Blank	Real water			
1.5	0.2	1.48	1.43	0.71±0.39	55	157
1.5	0.3	1.43	1.38	0.54±0.25	46	120
1.5	0.5	1.28	1.23	0.46±0.04	8	102
1.5	1	1.14	1.09	0.43±0.04	9	96
1.5	1.2	1.02	1.97	0.46±0.05	10	102

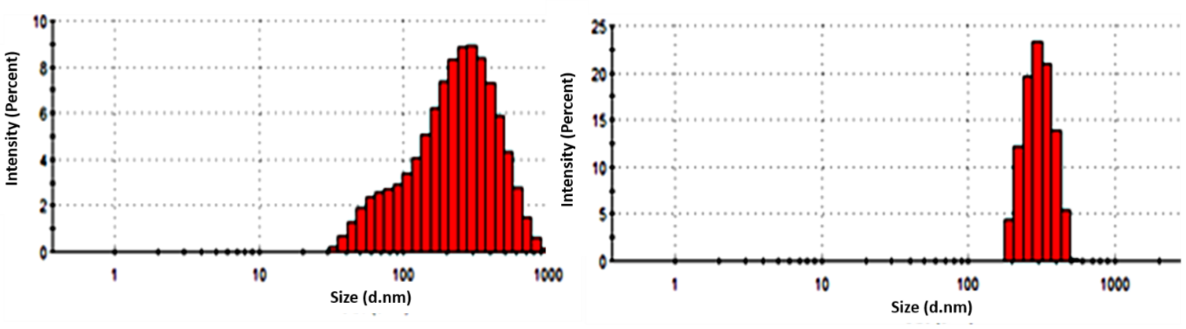

Fig. S10. Hydrodynamic size obtained from DLS analysis for equal volume (1:1) aqueous colloidal mixture of p-quinonimine and p-quinone functionalized AuNP at pH 7-8 towards addition of (a) 250 mL and (b) 500 mL aqueous solution of 10 ppm As(III) ions

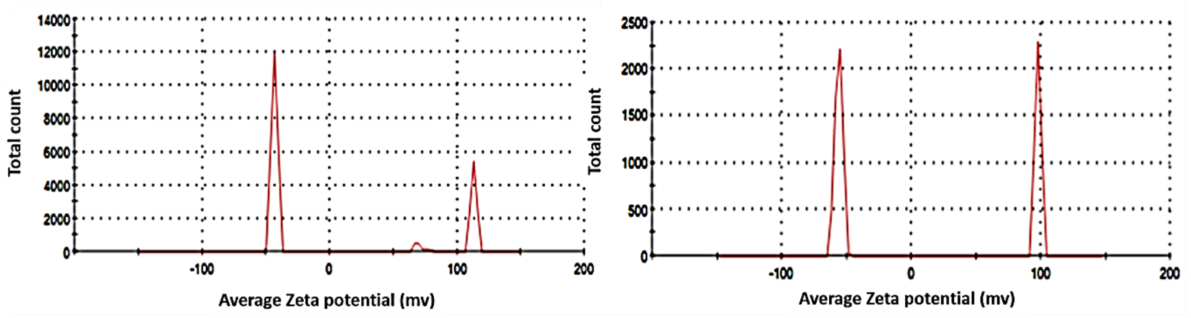

Fig. S11. Zeta potential obtained from DLS analysis for equal volume aqueous colloidal mixture of p-quinonimine and p-quinone functionalized AuNP at pH 7-8 towards addition of (a) 250 mL and (b) 500 mL aqueous solution of 10 ppm As(III) ions
